# Who Are Dentists?

**Published:** 1861-06

**Authors:** Wm. A. Pease


### WHO ARE DENTISTS ?—No 4.

BY WM. A. PEASE.

In resuming the consideration of the materials used as a base for artificial teeth, I shall devote this number to rubber or vulcanite, as the one next in order and importance. This rubber is nothing more than the common India rubber of commerce, with which every one is familiar; to which is added a certain proportion of sulphur, and the whole is then colored by mixing with it the *red sulphuret of mercury*, to make it faintly resemble the gum. After it is thus prepared, it is moulded on a model to fit the mouth; and it is then submitted to a high degree of heat or steam, in an apparatus designed for that purpose, to harden and give it firmness and rigidity.

Whether this material is susceptible of further improvement is a matter of speculation. As yet the color is objectionable; and there are doubts as to the propriety of putting so much sulphuret of mercury, as a fixture, into the mouth. In reference to it, it has been facetiously remarked, that it is good for the liver, whatever effect it may have upon the mouth and gum. Although rubber has been used quite extensively and with little discrimination for a year, and in a few experimental cases for four years, but few cases of irritation or ulceration of the mouth have been reported, and the history of them is so obscure that it is still uncertain whether they were caused by the coloring material, or from mechanical irritation. It is believed, however, that the danger of salivation is not great; but, with our present knowledge, no one knows, who uses it, but that his next patient may be affected, and present some of the symptoms of mercurial disease. As was to be expected from our experience with combs and other articles made of rubber, which, though in use for considerable periods of time, still retain the peculiar and offensive smell of the material, we find that sets of teeth mounted upon this base are equally odoriferous, although the wearer soon ceases to notice it.

Vulcanite as a base for artificial teeth is in form and general appearance, color excepted, nearly identical with sets on continuous gum or enameled work. In bulk they are about alike; both are filled in around the teeth so as to leave no interstices for the accumulation of food; and to both, the plumper can be added to give additional fullness to the face. Thus, in these particulars being equal, they furnish a base upon which to institute a comparison of their other respective qualities, as well as their comparative merits, with other materials.

We have before remarked, that one of the chief objections to sets of teeth on the platina base consisted in its weight, and the greater tax it occasions to the roof of the mouth. Rubber is not liable to this objection; as it is not only much lighter than platina, but it is lighter than sets of teeth on gold or silver. In this consists its chief excellence. Persons who have worn sets of teeth on other materials, after trying the vulcanite, speak of the comfort it affords them, in the absence of the weight and dragging down sensation, caused by other materials. But this want of weight makes it but illy adapted for sets of teeth for the under jaw, where weight is an advantage, and is desirable to keep the set in its place. For this purpose the heavy sets on platina would seem to be desirable; but it is found the greater changes constantly taking place on the under jaw from absorption, cause the pressure upon the set to be unequally distributed, which makes it spring, and the enamel to check, crack and flake off. Thus it will be seen that while the platina sets, from their great weight, are not as well adapted for the upper, we can not avail ourselves of the weight, which is desirable for the under jaw, without incurring the risk of frequent fractures and other injuries, requiring tedious and expensive repairs. On the other hand, the little weight of rubber makes it comfortable and agreeable on the upper, but it is liable to the same objections as platina, on account of the changes of the under jaw; with the additional disadvantage of greater mobility by reason of its small specific gravity. In comparison with these, sets on gold possess a valuable medium; the weight is not sufficient to severely tax the upper, and it is enough to give considerable stability to sets on the under jaw; and its elasticity is so great as to secure it from easy fracture.

Of the durability of rubber as a base for artificial teeth, but little can be said. It has been used experimentally and as a curiosity a little more than four years in a limited number of cases, and quite extensively for a year. When we consider that the average duration of sets of teeth on gold is at least six years, and, but for the large amount of work made by mechanics and poor operators, it would be much longer, it will be seen that the longest duration of any set of teeth on rubber has not been so great as the average duration of them on gold. Already the percentage of repairs is considerable, and some, who have had the most experience with the material, think, unless great improvements are made in it, its chief value will be but for temporary purposes. As remarked in another chapter, an important element of value in all dental substitutes is facility of repair. Sets of teeth on rubber are not easily repaired; and it is asserted that each successive repair so weakens them, that by the time they have been two or three times repaired, it will generally be cheaper, and often necessary, to make a new set.

Probably no material for dental purposes was ever brought so suddenly into use, or was used so indiscriminately as vulcanite or rubber. No other material was ever introduced or backed by so great and powerful a monopoly as the American Hard Rubber Company, which had a great interest at stake, not only in the sale of the material, from which it derived a profit, but also, from the license fee of one hundred dollars, which it exacted from every dentist who used it. No other material ever required so little skill to use it as this. It leveled all distinctions, made it possible for the merest tyro to fit a model, which before could only be done by experience and skill. Hence it was eagerly seized, extensively used, and advertised by persons of but little skill. They created a public opinion in its favor, and called all those who did not use it, old fogies—men who did not keep step with the march of improvement. Hence many were forced to use it by the circumstances by which they were surrounded, or because they were too selfish or too poor to be able to lose their position or patients for a doubt. In the intoxication of the moment, it was praised extravagantly,—it was called better than gold, and suiting the action to the words, gold was taken from the mouth and replaced with rubber. It was used in suitable and unsuitable cases; for the refined and vulgar; for the rich and poor; for those on whom the loss would fall lightly, if it proved to be valueless, and for those who invested their all in it, trusting that it would prove as represented, and be of life-long service. Thus it was used in a manner and to an extent wholly unwarranted by any knowledge dentists then had, or now have, of its value or durability. Already this exhilaration begins to subside—dentists begin to manifest some degree of alarm; they begin to inquire what will become of them? what will be their position if their apprehensions should prove true? and the more cautious and responsible are now using it less and with greater discrimination. Our opinion is, that it has been used altogether too much; that for the next two or three years it should only be used in cases eminently suitable for it, or for temporary purposes. If, after that time, it should prove to be valuable, the community will readily pardon what they will then consider a creditable excess of caution; but if it should prove to be of small value, the thousands of persons, who now have it in their mouths, will hardly forgive what they will then consider an unwarrantable and indecent haste in thrusting upon them a base material, which will then be odoriferous in more ways than one.

Thus, having passed in review the principal materials used as a base for artificial teeth, we shall close this chapter with a paragraph, in which will be enumerated some other materials that are sometimes used for that purpose. Such engage the professional and public attention for a brief period, like the butterflies of the season, and like them as suddenly disappear. There are now three materials that still have a nominal use; that yet linger on the outskirts of the profession, used as a desperate means of advertising, but which are too unimportant to demand serious attention. The first we shall notice consists of a combination of base metals, of which tin is an ingredient, which is cast upon the model. The combiner, in his greed to make a fortune out of dentists, took out a patent for this, for which he asked so much for the right to use it, that few would purchase; and it is probable, if he had given a free use of it to the profession by publishing the formula in the dental journals, as is usual with honorable dentists, its fate would have been the same. The other two materials belong to the plastic, or ceramic order, in both of which the base is molded on to the model like clay, of which one is composed, and they are then baked in a kiln like brick. They have never received much professional or public favor; their chief value having been to attract attention to offices, and gain for their makers a brief, but unenviable notoriety.

